# Synthesis and characterization of molecularly imprinted polymers for the remediation of PCBs and dioxins in aqueous environments

**DOI:** 10.1186/2052-336X-12-82

**Published:** 2014-05-09

**Authors:** Phumile Sikiti, Titus AM Msagati, Bhekie B Mamba, Ajay K Mishra

**Affiliations:** 1 Department of Applied Chemistry, Faculty of Science, University of Johannesburg, Doornfontein Campus, Johannesburg 2028, South Africa

## Abstract

This paper, reports on the approach devised to remediate water sources contaminated with PCBs and dioxins. The approach reported is based on the synthesis of highly selective molecularly imprinted polymers (MIPs). The paper elaborates the materials, procedures and protocols devised and followed for the synthesis of MIPs. The characterization of the synthesized MIPs and NIPs were performed using a number of techniques, such as FTIR, SEM, etc. The FTIR results show a broad OH stretching vibration peaks associated with methacrylic acid carboxylic group (COOH). at 3710 cm^-1^ for NIP and 3588 cm^-1^ for MIP, -CH_2_ stretching peak at 2953 cm^-1^for NIP, peaks due to the presence of methylene group in both MAA and EDMA appearing at 2951 cm^-1^ for MIP. The carbonyl group C = O stretching peak was observed in both MIP and NIP at 1721 cm^-1^ and this might have originated from MAA and EDMA respectively in both MIP and NIP. Weak combination bands from 1637 cm^-1^ to 1249 cm^-1^ and sharp bands at 1143 cm^-1^ specifically on MIPs spectra indicated the presence of aromatic ring of the template. The surface area of MIP was found to be 74.0010 m^2^/g, thus larger than that for NIP which was 58.6519 m^2^/g due to the presence of cavities on MIPs. The fit of the Langmuir model was found to be r ^2^ = 0.5842 while Freundlich model were found to be r^2^ = 0.3241, signifying that better correlation was with Langmuir than Freundlich.

## Introduction

Remediation of persistent organic pollutants (PoPs) at ultra-trace is always mandatory due to the fact that, many of the pollutants belonging to this class of compounds and which are found in the environments are toxic even at minute concentrations. There are many remediation methods and techniques for PoPs, but the majority are based on absorption. In the past, activated carbon emerged as a powerful method for remediation of pollutants from aqueous media [1]. But this technique is known to be inadequate to remediate many organic pollutants at low concentration levels [2]. Moreover, activated carbon applications are restricted due to its high cost especially where high quality activated carbon is needed.

Therefore in order to reduce the associated costs, many attempts have been made to find inexpensive remediation techniques for PoPs. Among the methods that were found to be attractive in terms of cost effectiveness is the use of silicates minerals such as zeolites. However, zeolites have got one limitation in that, they show low efficiency in the remediation of persistent organic pollutants [3]. Membrane techniques such as reverse osmosis have shown high capability and efficiency in terms of removing PoPs from aqueous media, but like high grade activated carbon, reverse osmosis is known to be expensive in terms of costs of operation and manufacturing [2].

The use of carbon nanotubes (CNTs) on the other hand have shown potential as remediation technologies for PoPs in water, but the costs associated with their production is a limiting factor [4]. The dioxins group of compounds comprise of 75 different chemical class of polychlorinated dibenzo-p-dioxin (PCDDs), and 135 polychlorinated dibenzofurans (PCDFs). They are known to enter into the environmental matrices from sources such as chemical and pesticides manufacturing industries, pulp and paper bleaching industries, burning of household’s trash, forest fires and burning of industrial and medical waste products [5]. While, polyaromatic biphenyls (PCBs) are comprised of 209 different chemical class of compounds, which were firstly produced due to their chemical stability, hence finding use in transformer capacitors, paints, printing inks and also in many other industrial applications [6]. These compounds also enter into the environment through the accidental spills and leaks during the transport of the chemicals or from the leaks on fires in transformer capacitors or other product containing PCBs [7].

However, out of 75 different chemicals class of dioxins, only 7 of them, 10 out of 135 furans, and 12 of 209 PCBs, are known to be highly toxic [8]. In addition, because of their low water solubility, hydrophobic character and resistance to metabolic degradation, these persistent organic pollutants (dioxins, furans and PCBs) are found in wide range of biological samples (e.g. adipose tissues), where they tend to bio-accumulate through food web [9]. Exposure to these molecules in animals and humans cause an adverse health effects including mutagenicity, carcinogenicity, reproductive disorders, immune suppression, birth defects and they are also endocrine disrupters [10]. Thus, there is need to develop an effective, yet economical methods for the removal of dioxins and PCBs at low concentration levels in environmental waters. More importantly, in May 2004, the Stockholm Convection resolutions on persistent organic pollutants entered into force with the intention of reducing or ultimately eliminating these pollutants. However, South Africa as part of this Convection is legally obligated to abide by the objectives of the Treaty and is thus expected to support research on persistent organic pollutants [11]. One of the attractive technologies that show potential for the removal of PCBs and dioxins in water is the use of molecularly imprinted polymers (MIPs). The important features for MIPs that are attractive include stability, low cost, ease of preparation, versatility and resistance for a wide range of pH and temperature [12-14].

**Scheme 1:**
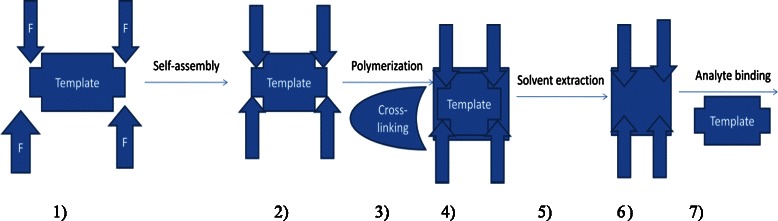
**Schematic representation of molecularly imprinted polymer synthesis using non-covalently interaction.** Where 1) f = functional monomer mixed with template followed by self-assembly by heating process. 2) Interaction between the template and functional monomer. 3) Polymerization and addition of cross-linking to get mechanically stabilize the polymer matrix - 4) followed by solvent extraction to remove template at step 5). At step 6) polymer matrix that complementary in shape, size. 7) The binding of analyte.

Molecularly imprinted polymers are man-made polymers with recognition binding sites which are able to bind a molecule of interest or its structural analogous from complex sample matrices [15,16]. The MIPs molecule is formed based on the polymerization of functional monomers and cross-linker molecules in a complementary shape around a template molecule. When the template is extracted or it is removed, it results into the formation of recognition binding sites in the polymer matrix, hence the template/target analyte of interests can be recognized from the complex environmental samples into that polymer matrix. There are several steps that are involved in preparation of molecularly imprinting. The first steps involve the generation of the template molecule which is to be mixed with functional monomer (units that finally react to form the polymer). These monomers have specific chemical functional groups such as carboxyl, hydroxyl, amino, or aromatic groups which can bind to the specific molecule covalently or non-covalently. In this work the non-covalently interaction will be adopted due to the suitable interaction between template and functional monomer. Another attractive feature for non-covalently protocol is that it easily conducted, avoiding the tedious synthesis of polymerization complex, and also great variety of functionality can be produced into molecularly imprinted by using non-covalently interaction methods. The second step in the MIPs synthesis involves the formation of polymerization assembly using excess of cross-linking agent to give a highly cross-linked, rigid, glassy polymer with functional groups fixed in a specific orientation around the template molecule. Then the embedded template is then chemically extracted from the polymer, creating a rigid imprint of the molecule inside the polymer matrix (Scheme 1).

The application of MIPs for removal of target analyte species or as sorbents has been reported for many organic compounds from different samples matrices, for example different MIPs for different analytes such as atrazine [17], Nitrophenol [18] sulfonylureas [19] have been reported. In addition to these, MIPs for remediation of dioxin have as well been reported [20]. Hence, the aim of this work was to synthesize and characterize molecularly imprinted polymers for the efficient remediation of polyaromatic biphenyl (PCBs) and dioxins from water samples.

## Experimental

### Materials and methods

Standards 2,3,7,8 TCDD and PCB-1 (2-monochlorobiphenyl), PCB-28 (2,4,4-tri-chlorobiphenyl), PCB-101 (2,2,4,5,5-pentachlorobiphenyl) and PCBs-77 (3,3′,4,5′-tetrachlorobiphenyl), and the organic solvents (AR grade) were purchased from Riedel-de-Haën (Seelze-Hannover, Germany). Pesticide quality solvents (n-hexane and chloroform) were purchased from Panreac (Barcelona, Spain). Chemicals for the synthesis of molecularly imprinted polymers (MIPs), which include, functional monomer methacrylic acids (100 g, 98% purity), porogen solvent chloroform anhydrous (1 L, GC grade 99%), Standard of dioxin (2,3,7,8 TCDD) (1 mL, 97.5% purity) which was used as a template, 1,1′^-^ Azobis (cylohexanecarbonitrile) (25 g, 98%) used as an initiator, cross linker ethylene glycol dimethacrylate (25 g, 98%), acetic acids (2.5 L, 100%), methanol (1 L, 99.8%), were all purchased from Sigma-Aldrich (St Louis, MO, USA). Nitrogen gas for purge was purchased from AFROX (Johannesburg, South Africa).

### Preparation of standards

PCBs stock solutions (1000 mg/L) and 2,3,7,8-tetrachlorodibenzo-p-dioxin (1 μg/L) were prepared in hexane and were stored at 4°C in a refrigerator. Fresh working solutions were prepared daily by appropriate serial dilution of the stock solution with hexane as a solvent.

### Preparation of molecularly imprinted polymers and non-imprinted polymers

In this work, molecularly imprinted polymers were synthesized via a bulk polymerization method, with 2,3,7,8-tetrachlorodibenzo-p-dioxin as a template molecule, methacrylic acid (MAA) as a functional monomer, ethylene glycol dimethacrylate (EDMA) as a cross-linker, chloroform as porogenic solvent and 1,1′-azobis(cylohexanecarbonitrile) (ABCHC) as an initiator.

In the procedure, different molar ratios of template to the functional monomer were prepared in order to get the best working molar ratio which was found to be 1:6. Therefore 1 mmol of 2,3,7,8-tetrachlorodibenzo-p-dioxin and MAA were dissolved in 6 mL of chloroform in a flask, and the mixture was left to stand for few minutes for prearrangement and then 32 mmol of EDMA and 0.103 g ABCHC were added. The prepared sample mixture was then degassed and purged with nitrogen before being sealed with a septum. The polymerization was carried out at 60°C for 24 hrs in a thermostated water bath. The obtained polymers were mechanically grounded into powders, followed by washing using a mixture of methanol and acetic acid (9:1, v/v) in a Soxhlet apparatus to remove the template molecule. The powders with particle sizes ranging from 56 to 74 μm were selected, washed, dried and stored in a desiccator. In a contrast experiment, the control polymer or the non-imprinted polymers (NIPs) were prepared using a similar procedure but without the addition of the template molecule, 2,3,7,8-tetrachlorodibenzo-p-dioxin.

### Instrumentation

The gas chromatograph model 7890A series (Agilent Technologies, Inc., Wilmington, Delaware, USA) coupled to a LECO Pegasus 4D time of flight mass spectrometer were used for all separations and detection of PCBs and dioxin extracts. Restek columns; Rxi-5Sil MS (30 m × 0.25 mm i.d × 0.25 μm film thickness) primary column and Rtx-200 (0.69 m 0.18 mm i.d × 0.1B film thickness) secondary column were used for all separations. The flow rate of the carrier gas (helium) was 1.5 mL/min and the injection type 100:1 split with volume of 2 μL. The injection temperature was set at 250°C and the oven temperature was programmed as follows: 35°C held for 0.25 minutes; ramped from 35°C - 120°C at 60°C/minute, then 120°C - 220°C at 80°C/minute.

The mass spectrometry conditions were set as follows: Ionization: Electron ionization at -70 eV; source temperature: 180°C; stored mass range: 47–350 um; acquisition rate: 20 spectra/second; detector voltage: -1500 V. For spectroscopic characterization, a Thermo Scientific (Nicolet IS 10) Fourier transform Infrared (FT-IR) was employed while thermogravimetric analysis was done using a Perkin Elmer TGA 4000. The surface morphology was analyzed by using scanning electron microscopy (SEM) from TESCAN WIRSAM scientific and precision equipment (PTY) LTD. The surface and porosity analysis of the synthesized polymers were performed using Brunauer, Emmett, Teller (BET) Micromeritics ASAP 2020 surface and porosity analyzer.

### Rebinding studies for PCB-77 compound

For the preparation of a calibration curve of PCB-77, solutions of standards (50-250 μg/L) were prepared by dilution of 1000 μg/L solution in hexane. Then in another separate experiment, 3 mL of (0.3-10 μg/L) of PCB-77 in which 3 mg of MIP and NIP were added separate vials. The solutions were then sealed and shaken in a shaker at 25°C for 24 hrs. The suspension was then filtered using 0.22 μm PVDF filter. The concentration of the filtrate was analyzed by using GC-TOF-MS. The amount of analyte bound to the MIP was calculated using Equation 1.

(1) $$q_{e}=V\;\frac{\left(\mathrm{Co}‒\mathrm{Ce}\right)}{m}$$

Where q_e_ is the amount of analyte adsorbed in an adsorbent (MIP or NIP), V is the volume of the solution, C_O_ is the initial concentration and C_e_ is the concentration of target analyte at equilibrium or after adsorption.

## Results and discussion

### FTIR characterization of MIPs and NIPs

**Figure 1:**
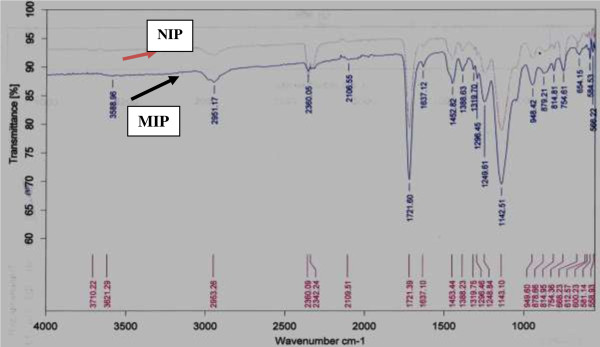
FTIR spectra of NIP and MIP.

Molecularly imprinted polymers (MIPs) and non imprinted polymers (NIPs) were prepared via bulk polymerization method using a non-covalent approach. The interaction between functional monomer and template molecule provides the affinity and high selectivity of MIP. In this study FTIR in Figure 1 was used to confirm peaks after co-polymerization had been done. The FTIR results show a broad OH stretching vibration peak at 3710 cm^-1^ for NIP and 3588 cm^-1^ for MIP. These peaks can be associated with methacrylic acid carboxylic group (COOH). The -CH_2_ stretching peak was also observed at 2953 cm^-1^ for NIP while the peak appearing at 2951 cm^-1^ can be attributed for MIP, and this can be due to the presence of methylene group in both MAA and EDMA. The carbonyl group C = O stretching peak was observed in both MIP and NIP at 1721 cm^-1^ and this might have originated from MAA and EDMA respectively in both MIP and NIP. Weak combination bands from 1637 cm^-1^ to 1249 cm^-1^ and sharp bands at 1143 cm^-1^ specifically on MIPs spectra indicate the presence of aromatic ring of the template.

### SEM characterization of both MIPs and NIPs

**Figure 2:**
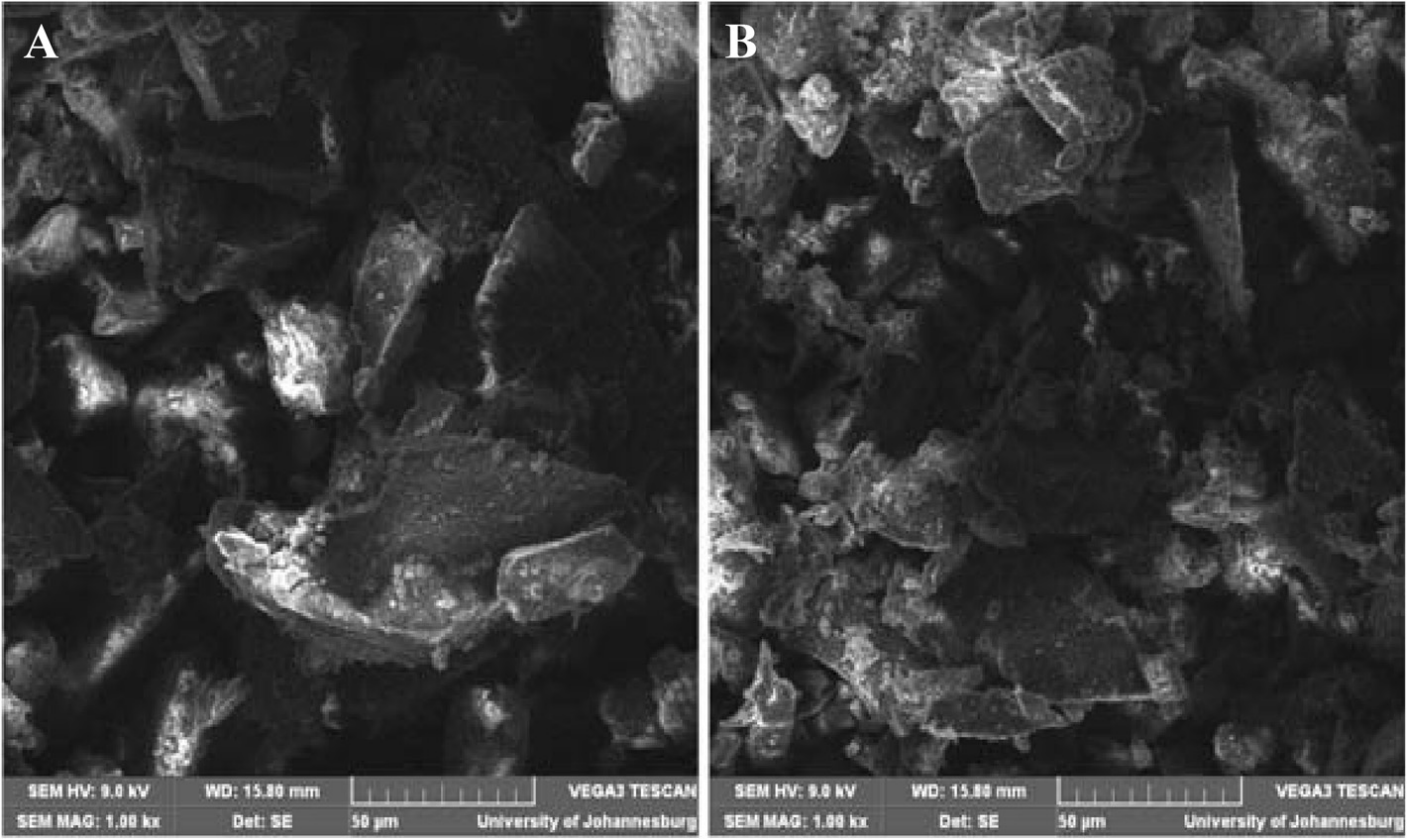
SEM micrograph images of A: = MIP and B: = NIP.

The surface morphology and the particle size of both MIP and NIP were analyzed using scanning electron microscopy (SEM). The results indicates that the nature of the particles obtained after preparation of polymers by bulk polymerization were all found to be more irregular Figure 2A and B. The control polymer (NIP) was observed to have smoother surface than the MIP, while the MIP after the template removal on the other side had rough surfaces (Figure 2) which can be attributed to the formation of cavities during the synthesis process. It has been reported that the roughness of MIP particles can lead to high surface area than that of NIP and thus MIP can adsorb analytes of interest much better than the NIP.

### BET characterization of the imprinted (MIPs) and the non-imprinted (NIPs) polymers

**Figure 3:**
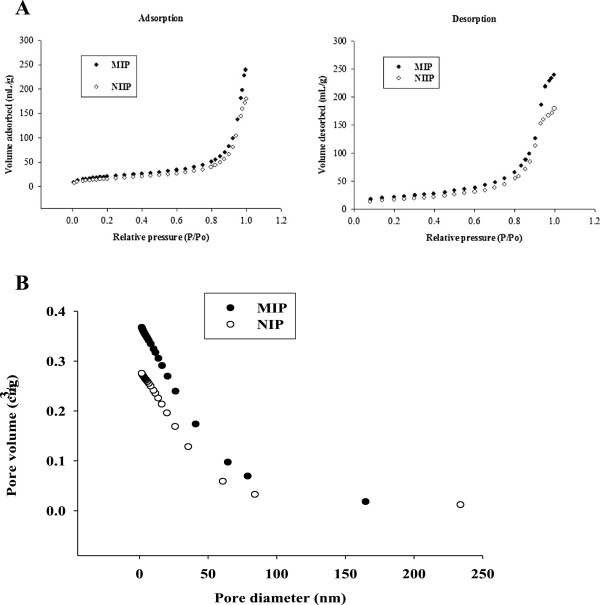
**HPLC glass vial containing an insert. A**. Adsorption of MIP and NIP, and desorption of MIP and NIP. **B**. Variation of pore volume versus pore diameter for MIP and NIP.

The adsorption-desorption isotherms of nitrogen (N_2_) at 75.6 K on the MIPs and NIPs are shown in Figure 3A and B which show the relationship between the amount of nitrogen adsorbed relative to pressure. The volume adsorbed of MIP and NIP increased with relative pressure followed by a sudden increase in nitrogen uptake at a relative pressure from P/P_O_ < 0.8 and this might be due to capillary condensation. However, both MIP and NIP adsorbs and desorbed. The surface area of MIP was found to be 74.0010 m^2^/g, thus larger than that for NIP which was 58.6519 m^2^/g (Table 1). This observation simply means that MIP had larger surface area than that of NIP due to the presence of cavities on MIPs. Moreover, the pore volume and pore size for MIP were also found to be larger than that of NIP. In this case the larger the surface area the larger the pore volume and pore size. This may be due to the presence template molecule, such that after its removal from the polymer, it left rough particles of the MIP with higher surface area.

**Table 1:** Surface area, pore volume, and pore size of MIP and NIP

**Polymer type**	**Surface area (m**^**2**^**/g)**	**Pore volume (cm**^**3**^**/g)**	**Pore size (Å)**
MIP	74.00	0.3704	200.2
NIP	59.00	0.2777	189.3

**Figure 4:**
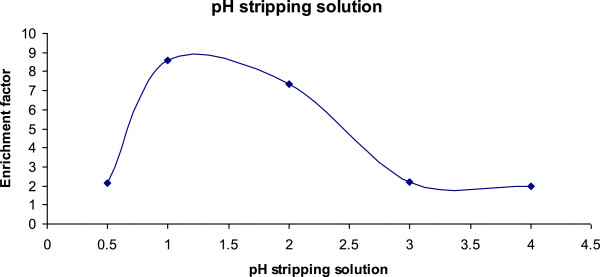
Acceptor pH optimisation for 1 mg/L carbofuran; membrane, 5% TOPO in isooctan, Sample pH = 11, extraction time = 30 minutes and stirring speed of 300 rpm.

The pore size distribution was investigated by observing the variation of collective pore volume with pore diameter as shown in Figure 4. The pore size ranged between 2–50 nm which indicated that the pores formed in both MIP and NIP are mesopores with few macropores above 50 nm in both MIP and MIP.

### TGA characterization of the imprinted (MIPs) and the control (NIPs) polymers

**Figure 5:**
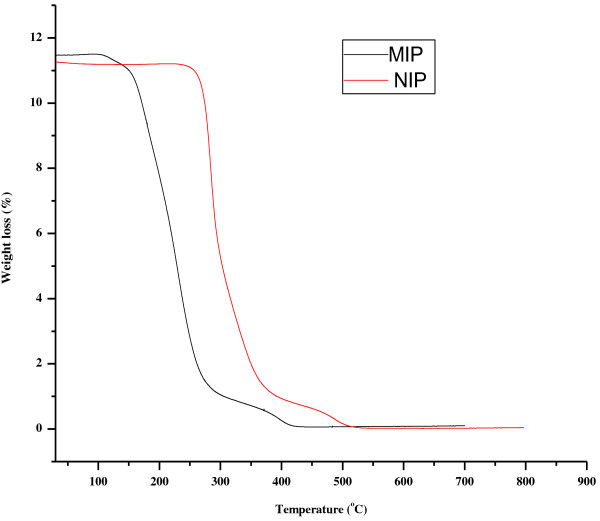
TGA decomposition curves of the imprinted (MIPs) and non-imprinted (NIPs) polymers.

The TGA plots of MIP and NIP are shown in Figure 5, whereby MIP plots show dehydration at temperatures of about 95°C to 100°C, while NIP dehydration is at about 50°C to 100°C. MIPs decompose gradually until 300°C then show stability from 400°C to 700°C. While NIPs a change is observed at about 350°C to 500°C and remain stable up to 800°C.

### Rebinding studies of PCBs-77

A series of standard solutions of PCB-77 ranging from 100 to 250 μg/L were prepared by serial dilution from a stock solution of 1000 μg/L. The linearity was shown from the standard curve by the line of best fit with r^2^ = 0.9995 for the equation, y = + 715.208x – 10333.9 as obtained from the graph. In addition the prepared concentrations were used for the binding studies for both the synthesized imprinted polymers (MIPs) and the control polymers (NIPs) whereby the binding was performed using solutions with concentrations ranging from 0.3 to 10 μg/L.

However, in order to find the binding amount adsorbed versus initial concentrations were plotted. The amount adsorbed is important parameter, as that gives a measure of the amount of PCBs in the adsorbent.

**Figure 6:**
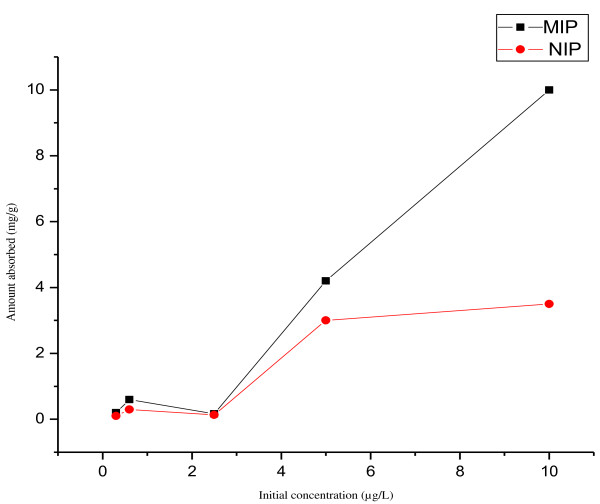
Comparison of adsorbed isotherm towards MIPs and NIPs.

In this study binding were carried out by varying the initial concentration of the 0.3 to 10 μg/L keeping other parameters such as volume of PCBs (5 mL) constant and mass of MIP or NIP (5 mg). The maximum binding capacity for PCB-77 in MIP was found to be 10 mg/g and for NIP was 3.5 mg/g. Figure 6 shows the binding of MIP and NIP. The increase of initial concentration leads to the increase in the binding capacity and at the MIP show a higher analyte amount that was adsorbed than that for NIP. This is because NIP binds PCB-77 only at the surface (physisorption process); while on the other MIP is binding PCB-77 using the cavities formed during polymerization (chemisorption) and the surface.

However the fit of the Langmuir model was found to be r ^2^ = 0.5842 while Freundlich model were found to be r^2^ = 0.3241. That show better correlation of the present data with Langmuir than Freundlich.

## Conclusions

The use of MIPs for the removal of specific compounds from loaded waters is advantageous due to the high level of selectivity the MIPs offer. Since the synthesis is simple and straightforward, the preparation of these polymers can be performed in the most economical possible way and the application of such polymers can result in high efficiency in removing pollutants from contaminated water sources. Another attractive feature is that, the technology does not need highly skilled personnel to synthesize these polymers as the procedure is simple and it doesn’t involve many steps. However, the optimization of the absorption parameters and kinetics parameters is in progress, hence that will conclude if the binding is controlled by monolayer or multilayer surfaces of MIPs towards PCBs and dioxin. In addition, the selectivity using analogues structural which are known to be persistent organic pollutant in the environment is also needed to be done.

## Competing interests

The authors declare that they have no competing interests.

## Authors’ contributions

PS, carried out all the labwork (experiments), TAMM, BBM, AKM took part in the design of the experiments, supervision of the project. All authors read and approved the final manuscript.
